# Italians locked down: people’s responses to early COVID-19 pandemic public health measures

**DOI:** 10.1057/s41599-022-01358-3

**Published:** 2022-09-30

**Authors:** Virginia Romano, Mirko Ancillotti, Deborah Mascalzoni, Roberta Biasiotto

**Affiliations:** 1 Institute for Biomedicine, Eurac Research, Affiliated Institute of the University of Lübeck, Bolzano, Italy; 2 Centre for Research Ethics and Bioethics, Department of Public Health and Caring Sciences, Uppsala University, Uppsala, Sweden; 3 Department of Biomedical, Metabolic and Neural Sciences, University of Modena and Reggio Emilia, Modena, Italy

**Keywords:** Social policy, Ethics

## Abstract

At the beginning of 2020, the widespread diffusion of SARS-CoV-2 rapidly became a worldwide priority. In Italy, the government implemented a lockdown for more than two months (March 9–May 18). Aware of the uniqueness of such an experience, we designed an online qualitative study focused on three main dimensions: daily life during the lockdown, relationships with others, and public health issues. The aim was to gain insights into people’s experiences of, and attitudes toward, the changes caused by public health measures implemented as a response to the COVID-19 pandemic. We conducted 18 semi-structured interviews with Italian residents. The interviewees were recruited through mediators using purposive sampling to obtain a balanced sample with respect to age, gender, education, and geographical residence. Interviews were analyzed through qualitative content analysis. The lockdown affected a variety of aspects of people’s life, resulting in a significant re-shaping of daily activities and relationships. These changes, which entailed both positive and negative aspects, were met with resilience. Even though public health measures were generally considered acceptable and adequate, they were also perceived to generate uncertainty and stress as well as to reveal tensions within the public health system. When tasked with imagining a scenario with saturated intensive care units and the need for selection criteria, respondents showed a tendency to dodge the question and struggled to formulate criteria. Media and news were found to be confusing, leading to a renewed critical attitude toward information. The findings shed some light on the impact of the lockdown on people’s daily life and its effects on relationships with others. Furthermore, the study contributes to an understanding of people’s reasons for, and capacity to respond to, emergency public health measures.

## Introduction

The severe acute respiratory syndrome coronavirus 2 (SARS-CoV-2) causes a highly transmissible acute respiratory disease (COVID-19), which can present severe symptoms leading to death. The first cases of COVID-19 were reported in Wuhan, China, at the end of 2019 (WHO, 2020a). The disease quickly spread worldwide and became a global threat. On January 30, 2020, the WHO declared the COVID-19 epidemic a public health emergency of international concern (WHO, 2020b), and on March 11, 2020, a pandemic (WHO, 2020c). On January 31, Italy declared a state of emergency for the following 6 months. Italy was the first western country to be heavily hit by the virus and to implement severely restrictive public health measures. The northern regions of Lombardy and some provinces of Piedmont, Veneto, and Emilia-Romagna—which were the most affected areas in Italy at the time—were classified as a “red zone” and were subjected to the most rigorous limitations on socioeconomic activities and movements (Ministero della Salute, 2020a). On March 9, 2020, Prime Minister Giuseppe Conte extended such measures to the entire country, without making any distinctions based on the seriousness of the emergency at the local level (Ministero della Salute, 2020b). All commercial activities, social activities, schools, and universities were closed. Only a few enterprises that were deemed essential—e.g., pharmacies, supermarkets, and newsstands—were open. People’s movements were forbidden except for reasons (e.g., health and work) proven by a self-declaration. With this, Italy entered ‘phase one’ of lockdown. Between February and May 2020, COVID-19 cases peaked, reaching more than 800 deaths per day in the last weeks of March (ECDC, 2020). The rampant increase in infections and deaths overburdened the healthcare system, which was facing an unprecedented need for intensive care units while suspending regular healthcare services. Phase one lasted until May 18, 2020. Subsequently, less restrictive measures were adopted—with a partial and gradual re-opening of non-essential services—following a decrease in infections and deaths during the summer period (ECDC, 2020).

Our research team realized that the lockdown, given its exceptionality, constituted a unique opportunity to conduct social science research. We decided to explore people’s responses to the circumstances in which they were living using semi-structured interviews with a sample of Italian residents. The present study aims to understand the impact of the public health measures in place in Italy following the COVID-19 emergency in spring 2020 on laypeople, and to explore their views on the measures and public health challenges associated with the health emergency.

The social impact of the pandemic and public health measures was investigated in several studies with different approaches and methods. Studies conducted in European countries with a similar focus on themes and data collection timeframe investigated mental health and the psychological impact of the lockdown, and the impact of lockdown on daily life (Ahrens et al., 2021; Martinelli et al., 2020; McKenna- Pérez-Rodrigo et al., 2021; Pieh et al., 2020; McKenna-Plumley et al., 2021; Probst et al., 2020; Schwinger et al., 2020,). In Italy, studies similarly aimed to understand the impact of the lockdown, focusing on mental health and everyday life disruption (Durosini et al., 2021; Ferrante et al., 2022; Risi et al., 2021; Tomaino et al., 2021; Trifiletti et al., 2022). Other studies focused on the perception of the public health measures, and linked risk perception to behavior and adherence to the measures (Atkinson-Clement and Pigalle, 2021; De Coninck et al., 2020; Liekefett and Becker, 2021; Lo Presti et al., 2022; Scholz and Freund, 2021; Savadori and Lauriola, 2021). Studies that included a wider timeframe in data collection grasped the impact of the lockdown and the response to public health measures over time (Marinaci et al., 2021).

In our work, we shared with some of the above-mentioned studies conducted in Italy the interest in the impact of public health measures in everyday life. Additionally, we provided a public health perspective by exploring the response to the measures and investigating views on public health management and challenges. The methodological approaches in studies conducted in Italy were wide, including for example semi-structured interviews, surveys, questionnaires, and diaries, but shared an “online” dimension, a shift in data collection which became necessary for empirical research conducted during a pandemic (Lupton, 2020). Our study, which was designed approximately a couple of weeks after the implementation of the lockdown, also took a remote approach to data collection.

## Methods

### Design settings

When we, as a research group, conceived and conducted the present study, we were all (except one author living in Sweden and helping us to anchor our views) experiencing the lockdown ourselves. By reflexively considering the genesis of our research (Berger, 2015), we were cognizant of the fact that our circumstances reflected the conceptualization of the study, the definition of the main research questions, and their operationalization in the form of a semi-structured interview guide. Single individual interviews were carried out during the lockdown when both the interviewer and interviewees were living in the same circumstances.

### Interview guide

The exploration of areas of interest started with brainstorming within the research team. After the brainstorming phase, each member of the team independently elaborated a defined number of questions. After collecting all the questions, two main dimensions were identified as reflecting the main tensions found in the experience of the lockdown and public health management of the epidemic.

1. Daily life during the lockdown: daily life changes and re-arrangements, advantages and disadvantages, changes in priorities, changes in social relationships, and perception of others.
2. Public health response and pandemic challenges: perception of the public health measures, hypothetical criteria for access to scarce critical care resources.

Each dimension was operationalized through its respective specific set of questions. We also included a question on media and information on the pandemic, which in our view was important for contextualizing the situation people were living in at that time. The final version of the interview guide included nine questions (Supplementary information S1). Follow-up and probing questions were used for clarification and elaboration. The interview guide was tested once and, as there was no need to make any changes, the material produced was included in the data analysis.

### Recruitment

The interviewees were recruited using purposive sampling, the aim being a balanced sample concerning age, gender, education, and geographical area of residence in Italy (North, Center, South and Islands). Recruitment occurred through mediators (Kristensen and Ravn, 2015), which were recruited through the researchers’ network via phone. Mediators were provided with an overview of the research aim and setting and were asked to suggest and contact potential respondents within their social circle. If prospective respondents showed interest in participating, mediators facilitated the contact between potential interviewees and the researcher in charge of the interviews. After potential respondents received all the relevant information on the study from the researcher and agreed to participate, a video call appointment was fixed for the interview. Recruitment occurred via phone call.

Exclusion criteria were working in healthcare and self-reported positivity (at the time of the interview or earlier) for COVID-19. We considered that, in both cases, perceptions of the entire situation might have been greatly influenced by direct contact with the virus, thus constituting a special and specific experience. Trying to understand that experience was beyond the scope of the present study.

### Data collection

One of the authors conducted 18 interviews between April 27, 2020, and June 6, 2020, by video call. Approximately half of the interviews were conducted during phase one of the lockdown. The others were conducted during phase two. The average length of the interviews was 40 min. The interviews were conducted in Italian. The sample size was chosen based on saturation.

### Ethical aspects of data collection

In Italy, in the absence of legislation requiring the ethical approval of social science research, our research was not applicable for submission to an ethics review board. Our work conduct was inspired by the principles expressed in the Declaration of Helsinki and in accordance with relevant guidelines and regulations. It was also informed by the code of conduct for professional sociologists (http://www.societaitalianasociologia.it/p/codice-deontologico.html). Participants were informed about the project aim and rationale, the data treatment, that their participation was voluntary, and that they could withdraw at any time. All the information was provided orally, and participants provided informed verbal consent to participate. For further details, see the “Ethics approval” section.

### Data analysis

Interviews were audio recorded and transcribed verbatim. Transcripts were analyzed through qualitative content analysis (Hsieh and Shannon, 2005). After each interview, the research team discussed together impressions and preliminary themes and evaluated the efficacy of the interview guide (no adjustments were made throughout). All the authors read and became familiar with the content of the interviews. We used the dimensions and the interview guide as a structure for organizing the coding of the transcripts. Along the same line, the results are presented according to the dimensions or specific questions. The analysis was conducted on the Italian transcripts. Selected excerpts were translated into English for incorporation into the manuscript as representative quotes.

## Results

A total of 18 respondents participated in the study. Their sociodemographics are described in Table 1.

**Table 1:** Sociodemographic data on the respondents.

Sociodemographic characteristics	*N*
*Gender*	
Female	7
Male	11
*Age group*	
25–34	5
35–44	7
45–54	1
55–64	3
65–74	1
75–84	1
*Education*	
Higher education	6
Secondary education	12
*Area*	
Center	7
North	6
South and Islands	5
Total	18

### Daily life during the lockdown

The lockdown caused major changes in the participants’ life. It affected multiple aspects of their existence, from the most trivial daily routines to a redefinition of their relationships in the family and with others.

#### Impact

When asked about the impact of lockdown and public health measures on their daily routine, most respondents described a process including a phase of understanding and acceptance of what was going on followed by progressive development of and adjustment to a new routine. This was generally associated with a coping strategy that was useful not only in practical terms to keep things going but also in managing stress and anxiety. Though most respondents experienced initial difficulties in accepting the changes, they also reported adapting to them relatively quickly:

Q1. Right after the lockdown, everything went upside down because I went from work, work, work to home, home, home. In the beginning, I was frightened by this but, set aside the economic worries… I enjoyed it a lot because being used to organizing and planning, to be very systematic, I created a new routine for myself. Because not having a routine was a bit frightening for me I kept setting my alarm at the same time as always, I have a dog and I kept walking it at the same time, I exercised regularly, as far as possible. (Int. 17).

Work routines and the need to rearrange them followed the same path as the extra difficulty of transforming the spaces needed to work from home. For most respondents, working from home was perceived as a chance to spend more time with the family and on hobbies, as well as to have a less stressful lifestyle overall. Some respondents also indicated that the limitations on in-person social interaction resulted in stress relief. Most respondents experienced having more time (e.g., travel/commuting time):

Q2. This has clearly cut down my need to move around to zero, which given what I do is my most frequent activity and, as a consequence, it also solved my stress of having to move from one side of the city to the other and gave me more time to do “office” stuff. Another positive thing is that I got to spend more time with my daughter because clearly, with her being home from school and me spending the morning at home, she would often exercise with me, and it was a nice way to spend some time together. (Int. 16).

#### Relationships

The public health measures and new rules enforced to control the spread of COVID-19 polarized people’s behavior and feelings into a clear-cut distinction between what is acknowledged as “us” vs. “them.” Respondents admitted that they had both observed others’ behaviors and blamed others for not respecting the rules; they had also been criticized in the same way by others:

Q3. Look, I always respect rules, I get very annoyed when people don’t respect them, and I’m even more in this phase. Because if it’s compulsory to wear a mask, then it’s compulsory to wear a mask, if visiting relatives is not allowed, then you can’t visit relatives, while what you see is people going from one house to another, exchanging kids from one house to the other to do homework together and they tell you, it’s ok because we don’t have Coronavirus! Right! And how would you know?! (Int. 7).

Q4. Something that really annoyed me was the people who are called “balcony sheriffs” because, even if you were just going to take out the trash and you were maybe walking 100 meters, they would scream at you that you should have stayed at home. I thought this was really too much, I think we’re all closely following the rules and that strolling was cut to the minimum, just to get some fresh air. (Int. 16).

Somehow, the pandemic allowed people to publicly show their disappointment with or overtly criticize others. Social control was therefore experienced either actively or passively by all respondents. According to respondents, what also changed dramatically was the tendency not to trust other people’s ability to engage in responsible behaviors. In some cases, these accusatory attitudes were understood and tolerated as being a result of people’s fear, but, in general, most respondents were disappointed because they saw the emergency as a chance for society to change for the better, to incentivize solidarity and tolerance, something that, in their view, only occurred to a limited extent.

Q5. Coronavirus created an “us” and a “them.” A very distinct “us” and “them,” and this “us” and “them” is even more fragmented than it was before. And by “us” I really do mean my home, and “them” is everybody else, so you’re ok only if I know you, not even my neighborhood anymore… While in the first weeks we were all close together, we all loved each other, now this thing just blew up into a thousand pieces, and it’s a community that needs to rebuild itself. (Int. 15).

Aside from affecting relationships with others, most interviewees also admitted that the pandemic situation impacted their closest relationships. Many reported a negative change in their perception of close relationships, newly discovered attitudes that made them uneasy, and a general lack of openness in discussions and communication. This caused them to reflect on the meaning of friendship and to rethink what is important in meaningful relationships with friends.

Although less frequently perceived than the negative aspects, some respondents also talked about the positive consequences of the situation, such as a heightened sense of humanity and solidarity visible through tangible acts, offering an occasion for people to show strength and resilience. The use of a face mask was positively referred to as an act of respect for others and not perceived as a mere limitation.

### Public health

The lockdown and other public health measures generated different reactions among the participants. In this section, participants’ perceptions of and views on the measures are described, together with the scenario of overloaded intensive care units.

#### Opinions on public health measures

Among most of our sample, the general perception and overall evaluation of the measures implemented to control the pandemic were positive. Respondents expressed various reasons for supporting their positive attitudes that ranged from considering the measures adequate to stronger expressions of support. In the latter case, the measures were described as indispensable, rightful, and timely as well as needed to promote the common good; in other cases, respondents also indicated that, in their opinion, the lockdown should have been implemented earlier, thus showing even greater support for this containment strategy:

Q6. Well, the beginning was very bad because we had all reasons for the lockdown to be done a lot earlier…. I think that at the very beginning only Almighty Money was in control, and still is now, to change the topic, I heard that they want to restart Series A *[the Italian national soccer championship, Ed]*, and I think this is just nonsense, only linked to economic interests and not to public health factors, because we don’t care if they can or cannot play football, it’s just a game. (Int. 7).

The interviewees also shared their negative emotional response to these measures, a response that was filled with uncertainty, fear, and confusion. The latter was often linked to possible misunderstandings and perceived lack of clarity concerning what was or was not allowed as well as what constituted the right behavior in different social situations. For instance, in some cases, respondents found it difficult to clearly understand whether they were or were not allowed to go to work, and this uncertainty generated feelings of frustration. The limitation itself was not as stressful as the impossibility of clearly understanding the actual situational applicability.

Restrictions were often described as being in opposition to economic interests and the need to maintain productivity. Respondents often mentioned lobbying mechanisms of various kinds that promoted either postponing the lockdown measure or speeding up the re-opening of economic activities.

In the respondents’ opinion, the pandemic has brought to light the need to restructure the national healthcare system. In their view, the COVID-19 health emergency has challenged an already strained healthcare system and the management of healthcare resources. The scarcity of resources and the lack of coordination and preparedness between the regional and national levels impacted the efficacy of the pandemic response.

The pandemic also raised awareness of inequalities. Different socioeconomic and working conditions—exemplified by house size, proximity to nature and outdoor spaces, the ability to work remotely, and an unaffected regular, stable income—were perceived to inevitably affect the extent to which restrictive public health measures were bearable.

#### ICU criteria

It was admittedly difficult for respondents to express themselves about what criteria should be used to select which patients would be included in/excluded from intensive care units (ICUs). Whether they properly answered the question or tried to dodge it, one common mechanism was to distance themselves from determining in advance something that was not their decision to make. Concerns were voiced about who should make such life-or-death decisions, including the opinion that no one should. Typically, the issue was deemed to be an ethical dilemma that should ultimately be resolved by healthcare professionals. Attempts were made to answer the question by opposing its premise, such that the scenario was improbable or, on the contrary, realistic, and therefore efforts should be made to prevent it.

Respondents identified as possible selection criteria age, survival likelihood, role/function in society, and previous behaviors toward the collective. Concerning age, respondents stated that younger people should be prioritized:

Q7. It’s a matter of life expectancy, it’s not that the value of an elderly person’s life is lower, but if we evaluate based on how much one has lived, obviously the young person should be given the opportunity to live more than a person who has already lived a long time. (Int. 10).

Concerning role/function in society, healthcare professionals and researchers were pointed out. Behaviors against the collective were mentioned as potential exclusion factors, e.g., lockdown transgressors or perpetrators of serious crimes. Nonetheless, concerns were raised that it would be unfair to prioritize some groups over others or to place one group at the bottom of the list. The age criterion was specifically rejected in some instances, based on relational and sentimental grounds:

Q8. Yes, they are indeed elderly people, but they are still our grandparents, our uncles, and our parents, so even if they’re grown up, it’s not right that we should no longer have them with us (Int. 11).

Social class or strata were also mentioned as factors that should not be considered as criteria when deciding who should have access to intensive care.

### Information and media

Respondents’ experience of the lockdown and their view on the public health measures were inevitably affected by the information they had about the ongoing situation. Most of the respondents used TV, the internet, social media, and online newspapers as their main sources of information. Respondents also consulted other sources of information such as press releases, newspapers, radio, official government websites and resources, medical doctors, virologists, experts (in this category, both public figures, and personal contacts), journalists, and local and international news. Most of the respondents shared a feeling of exasperation with TV and the media in general during the pandemic: Information was found to be sensationalistic, and the media were perceived as lacking objectivity, amplifying the problem, and providing confusing and contradictory information, thus causing an increase in anxiety and a general lack of trust in media and information.

Q9. My trust in the communication and information system is decreasing…. That’s because you don’t know where to turn and can’t be certain you are turning to someone who can tell you something reasonable, correct, or useful…. You hear one person and then another who says exactly the contrary, you read one thing and then something else that again says the opposite. This has been difficult to accept and, moreover, it makes the search for correct information difficult. (Int. 8).

Respondents pointed to the importance of having a critical attitude toward the media, and they evaluated the reliability of the media and news by consulting and comparing more sources.

Q10…. seeing the news, the first question one asks oneself is: OK, who said that? Where does this news come from? So, now that the news is no longer considered reasonable a priori, we check to see if someone else says something afterward. Now, it’s like this: there is [a piece of news], is it valid? Is it not? Who is saying that? What’s the source? Show me that it’s true. (Int. 15).

## Discussion

### Reshaping daily life as a process

Respondents in the present study reported on the lockdown as a process more than something static; they described how puzzling the situation was and the whole process of progressive adaptation to such a complex and unprecedented situation. The need to talk about the lockdown in a complex and dynamic way justifies and supports our methodological choice of in-depth interviews. Respondents described how they had realized what was happening and coped with the situation by making a series of progressive adjustments. They reported strategies they had used to rearrange both their time (work and leisure) and their spaces (where to work, where to live), and through these changes, they unveiled their personal sensemaking tension in relation to the pandemic (Angeli and Montefusco, 2020).

One of the most striking social effects of COVID-19 was the polarization between “us” against “them,” characterized by the need to identify categories one can blame. As repeatedly reported by the respondents, during the Italian lockdown, the positive and communal feeling associated with everyone being together in an extreme and unprecedented situation was quickly followed by a heightened search for someone to blame. Some of the interviewees reported feeling this strong social control over themselves and being the object of shame, even when they were not breaking the rules. “Covidiots” is the new term used for people who find it hard to adhere to the rules because they are either “too weak, too stupid, or too immoral to do the right thing” (Reicher and Drury, 2021). “Pandemic fatigue” has also been associated with this uneasiness with rules, a general tendency to get “tired” of them, and, at the same time, negative feelings about those who break them (Michie et al., 2020). This same attitude of blame permeated interactions on social media, here with even more strength (Choli and Kuss, 2021). This narrative of blame leads to the bitter idea that COVID-19 presented as a “missed opportunity” for humankind to be better and do better. Nonetheless, the one clear positive effect COVID-19 had on social relationships was to strengthen those that were already valuable, and to cherish and underscore their preciousness.

### Complexities in public health

Complexity and adaptation were also described in the process that brought governments to policymaking surrounding the pandemic. Sensemaking in complex situations appears to permeate society at various levels, from the individual psychological one to the collective and political dimensions of regulations and emergency management (Angeli and Montefusco, 2020). The measures enforced in Italy to curb the spread of infection in the country were generally well accepted as necessary. Positive reactions to the need to implement anti-COVID-19 rules were also reported in other countries (Alanezi et al., 2020; Meier et al., 2020). The general acceptance of mitigation measures is connected to the general public perception of the risks associated with the spread of the COVID-19 virus (Motta Zanin et al., 2020). Where risk was perceived as stronger and mortality was higher, such as in the UK, Spain, and France, “the highest adherence (to rules) was reported” (Alanezi et al., 2020). Economic interests were often perceived as a potential obstacle to implementing measures that were in the best possible public interest as if there were a conflict between the righteousness of the measures and the survival of the economic system. In the literature, it has been estimated that the best possible solution for both public health and the economy appears to be “a prudent opening… whereas costs are higher for a more extensive opening process” (Dorn et al., 2022). What these measures clearly showed was the existence and exacerbation of socioeconomic inequalities in the population. As noted by Carta and De Philippis (2021), “the economic repercussions of the COVID-19 shock impacted low-income households more heavily than higher-income families, implying a substantial increase in labor income inequality.” The increase in inequalities tended to affect pre-existent fragilities both on a microsocial level, e.g., gender issues, and on a macrosocial level, i.e., developing countries over the so-called first world (Meraviglia and Dudka, 2021). In other words, COVID-19, which was initially called the ‘great leveler,’ actually turned out to expose ‘the fault lines in society’ and amplify inequalities at many different levels (Marmot and Allen, 2020).

The scenario of saturated ICUs leading to available resources only being allocated to some patients who may benefit from life-sustaining treatments was deemed improbable by some respondents. In general, it was a difficult issue to discuss. Even more problematic was specifying criteria for the allocation of scarce resources, i.e., how to choose who to treat and who to exclude from intensive care. Albeit problematic, the scenario was not at all improbable. Indeed, already on March 6, 2020, the Italian Society of Anesthesia, Analgesia, Resuscitation and Intensive Care (SIAARTI) issued a series of recommendations and ethical considerations to help clinicians involved in the care of critically ill COVID-19 patients in settings marked by scarce resources (Vergano et al., 2020). The opinions of the respondents in the present study were not too distant from SIAARTI’s recommendations, especially considering the *Triage principles and criteria*:

Age, comorbidities, and the functional status of any critically ill patient should carefully be evaluated. A longer and, hence, more “resource-consuming” clinical course may be anticipated in frail elderly patients with severe comorbidities, as compared to a relatively shorter and potentially more benign course in healthy young subjects. The underlying principle would be to save limited resources which may become extremely scarce for those who have a much greater probability of survival and life expectancy, in order to maximize the benefits for the largest number of people. (Vergano et al., 2020).

Lay people’s opinions and SIAARTI’s recommendations converged on the notion that age is a decisive criterion for ICU admission, but on different grounds. Although torn, most respondents seemed to apply a sort of fair innings argument, i.e., the view that there is some span of years that is reasonable for a person to have lived and if a decision must be made concerning whom to save, the life of the younger person should be prioritized. This view may easily be considered ageist, but appears to be supported by commonsense morality (Bognar, 2015). SIAARTI’s recommendation was justified by a maximization principle based on medical considerations. It is noteworthy that SIAARTI’s recommendations also stressed the importance of informing the patients and/or their proxies about the extraordinary nature of the measures in place, including the decision-making process behind withholding or withdrawing life-sustaining treatments, due to a duty of transparency and to maintain trust in the healthcare service (Vergano et al., 2020). In fact, the lack of transparency and the fear that some groups could be unfairly privileged constituted an often-discussed matter of concern in the interviews. The groups whose prioritization could be tolerated included healthcare professionals and researchers. This should probably not be interpreted as a form of compensation for their exposure to higher risks of infection or the heavy burden of their work, but instead, for the role that these categories of people could play in shortening the sanitary emergency and saving as many lives as possible. At the other end of the spectrum, among the groups mentioned that should be given the lowest priority, were lockdown transgressors and perpetrators of serious crimes. The answer to the normative question about ICU selection (or resource allocation) criteria could be derived by leveraging the values of utilitarian, egalitarian, and prioritarian approaches (Yuk-Chiu Yip, 2021). Most respondents swung between the former and the latter. The principle of distributive justice, intended as equality of access to finite health resources, did not appear to inform respondents’ views. Although previous studies on lockdown transgressors highlighted the fact that individuals considered their own (mis)behavior morally problematic, they also had different neutralizing strategies against feeling guilty (Cullen et al., 2021; Márquez Reiter, 2021). Nonetheless, people who were compliant with lockdown rules seemed to pass negative judgments on transgressors.

### Navigating information

During the COVID-19 pandemic and the imposed lockdown, information technology, and digital media acquired prominent importance in people’s lives, not only as a source of information on the pandemic, but also as a tool to work, learn, teach, connect with others, and engage in many other activities remotely, while living at home in a digitally connected world (Feldmann et al., 2021; Tropea and De Rango, 2020). During the COVID-19 pandemic, a massive amount of information, as well as misinformation, spread through the media, generating a so-called “infodemic” (Banerjee and Meena, 2021). The answers that respondents gave on media and information topics provided evidence of the key role trust played in their attitudes toward and relationship with information and media. Respondents in the present study seemed to be seeking strategies for a critical attitude that would allow them to navigate the overflow of information during the pandemic. They looked at media and information with a renewed critical lens: The information provided during the pandemic was often perceived as confusing and sensationalistic, thus leading to the development of strategies for analyzing sources’ trustworthiness, but also to a general lack of trust in media and information, which was also reported elsewhere (Van Scoy et al., 2021).

### Understanding change

To interpret the changes in people’s mindset and trust during the pandemic and the lockdown in relation to the observed information, and the changes that people faced as a result of the public health measures in place, we used the mindsponge mechanism framework (Vuong and Napier, 2015). The framework includes a multi-filtering information process and an inductive attitude that offers an understanding of the processes at stake in the change of mindset, cultural values, and identity. It seeks to understand “how an individual absorbs and integrates new cultural values into her/his own set of core values and the reverse of ejecting waning ones” (Vuong and Napier, 2015). It should be noted that information processes and decision-making during the COVID-19 pandemic were in the context of a life-and-death situation. Survival pressures (including social survival) had been driving information processes (Vuong, 2022) that shaped policymakers’ decisions (issuing the lockdown) and citizens’ responses (making behavioral adaptations). It is in such pressured conditions that the ideas of lockdown and adaptation were deemed valuable on both a societal and individual level.

The situation that the population in Italy had to face during the early pandemic phase and the lockdown is comparable to a change in cultural and societal values and context. Public health measures were adjusted according to the progressive acquisition of knowledge on the virus and the disease, and based on a daily assessment (e.g., the impact of the pandemic as the number of affected individuals, deaths, resources, etc.) of the worldwide and the local (at a national, regional, city-specific) context. At the same time, the public health care system struggled to meet the demand of people in need. The lack of adequate resources (e.g., facilities, structures, ICU departments, suitable protective gear) meant that, while the number of cases increased, the lack of intensive care units was a daily issue, entire hospitals were reconverted to COVID-19 departments, and all the other care services were suspended. Standardized monitoring and diagnostics systems were still under development and dependent on limited knowledge at the time. Individual behavior, social interactions, and movements were all regulated through norms that frequently left shaded areas in applicability and understanding. During the early phases of the pandemic and the lockdown, the public discourse that framed the implementation of the public health measures leveraged specific values: responsibility, courage, and sacrifice (Palazzo Chigi, 2020a, 2020b, 2020c, 2020d). The imposed and advocated changes in habits and lifestyles were framed as a collective effort for public health and the protection of all, especially those most vulnerable. National unity and pride were recurrent themes in the speeches of Prime Minister Conte when addressing the public to comply and cope with the imposed restrictions. The motto “Io resto a casa” (I stay at home) embodied all the above-described new public values emerging as a response to the national health emergency.

The changes that respondents reported in our study can be interpreted as the results of a filtering process, which consists of a dynamic integration and evaluation of information and new values. Against this background, the development of new routines can be interpreted as the beneficial result of a mindset change in managing stress. Expectations towards others and society were rethought and re-evaluated based on the perceived polarization of values of solidarity and tolerance vs. attitudes of blame, distrust, and social control. Both close relationships and social relationships were affected by the new mindset. An evaluation of cost and benefit intervened in the evaluation of the public health measures, perceived as important and necessary, but also stressful and framed in uncertainty. Respondents’ responses to the measures can be understood within a mindset change that included an increase in awareness of public health challenges and social inequalities. Trust played a major role in the filtering process of the information received by the media. Respondents elaborated on trustworthiness criteria to evaluate the news, and this process was the result of a renovated (mostly negative) perception of media.

Other studies with a focus on responses to the early public health measures implemented in Italy during the early pandemic phase, and on themes related to those we analyzed in our work interpreted change in everyday life, behavior, and emotional and psychological response. They achieved this through a theoretical framework that connected the effects of the macro-level change (referring to concepts of risk society) to the micro-level experiences (referring to the concept of framing) (Risi et al., 2021); personal construct theory framework (Tomaino et al., 2021); and Semiotic-Cultural Psycho-social Theory (Marinaci et al., 2021). Using the mindsponge mechanism framework allowed us to introduce a novel theoretical approach to the study of social change as a response to public health emergencies where a fast-paced development of information occurs, and impactful and restrictive public health responses are required.

### Limitations and strengths

Being explorative, the present study does not have any aspiration to be representative or generalizable to the wider population. We believe that the main value of the present work is to provide a privileged look at people’s experience of the earliest public health measures implemented in Italy during the outbreak of the COVID-19 pandemic. Both the study conception and the data collection occurred during the lockdown, allowing us to capture what it meant to experience the lockdown while experiencing it ourselves. This work may be seen as a starting point for further investigations into the social impact of the lockdown as a public health emergency measure that caused changes and restrictions in all aspects of people’s life. Although the process of changing mindset takes time, and the time frame considered in this study is relatively short (data collection occurred around two months after the beginning of the lockdown), we noticed that respondents reported changes, related to the experience of the pandemic and of the public health measures. Long-term changes at the individual and societal levels will be appreciated with further studies.

## Conclusions

The analysis conducted on the response to the public health measures implemented in Italy in February–June 2020 allowed us: to shed some light on the impact of the lockdown on people’s daily life and relationships with others; to explore views on the measures and on the problem of limited access to ICU during a public health emergency and the dilemma it creates; to grasp the transformed attitudes toward media and information. The insights obtained, which captured people’s responses to the earliest measures implemented in one of the most affected countries worldwide during the first wave of COVID-19, will be valuable for public health and emergency preparedness in possible future health emergencies because they highlight the changes in the social dimension caused by public health responses to health emergencies. Public health policy-making and planning may benefit from a qualitative study such as the one we conducted because it contributes to revealing people’s attitude and response to policy, and, in turn, anticipate or justify or contextualize policy success. Indeed, social aspects and public views are key to policy success (Vuong, 2018). By interpreting social changes as processes within the infosphere (for instance, through the information processing approach (Vuong and Napier, 2014)) and then analyzing the information inputs, policymakers and researchers could make assessments and effectively develop policy. As exemplified in the understanding of the COVID-19 vaccine production process (Vuong et al., 2022), a theoretical approach to the interpretation of change and innovation in relation to information may be useful for the management of health emergency responses and relevant public health policy design.

## Supplementary information

Supplementary

## Data Availability

The data that support the findings of this study are available from the corresponding author upon reasonable request. The data are not publicly available due to privacy or ethical restrictions.
