# Supplementary material for: Italians locked down: people’s responses to early COVID-19 pandemic public health measures
Source: Humanit Soc Sci Commun. 2022 Sep 30;9(1):342. doi: 10.1057/s41599-022-01358-3 (PMC9524326; doi:10.1057/s41599-022-01358-3)
Supplement: Supplementary file 1 — Supplementary [file 41599_2022_1358_MOESM1_ESM.docx]

## Supplementary information S1

### Interview guide

1. How has your daily life been changing during the lockdown?

2. Could you describe how you re-arranged your daily activities?

3. What are the major difficulties and disadvantages? And the advantages?

4. How did your priorities change?

5. How and which media do you use to get information on the emergency?

6. In comparison with the pre-pandemic situation, has your perception of the others changed? In which way (positive/negative)? Could you explain how/why and provide examples?

7. Do you believe that the public health measures implemented to limit the spread of the virus are/have been adequate? Why?

8*. Are you informed about the possibility that soon the government may use mobile phone data to trace people’s movements, as a strategy to deal with the pandemic (app Immuni)? Did you understand how this will work? What do you think about that?

9. This emergency has been overloading our health care system. In the hypothetical situation where the intensive care unit did not have enough capacity to admit all those in need, which criteria should be considered to decide who could access and who could not?

* The data obtained from this question will be analyzed elsewhere, together with other relevant data on the contact tracing app collected in the context of another study.
